# Antibacterial Derivatives of Marine Algae: An Overview of Pharmacological Mechanisms and Applications

**DOI:** 10.3390/md14040081

**Published:** 2016-04-22

**Authors:** Emer Shannon, Nissreen Abu-Ghannam

**Affiliations:** School of Food Science and Environmental Health, College of Sciences and Health, Dublin Institute of Technology, Cathal Brugha Street, Dublin D01 HV58, Ireland; Emer.shannon@dit.ie

**Keywords:** marine antibacterial, seaweeds, micro-algae, nutraceuticals, antibiotic-resistance, food preservation, disinfectants, allelopathy

## Abstract

The marine environment is home to a taxonomically diverse ecosystem. Organisms such as algae, molluscs, sponges, corals, and tunicates have evolved to survive the high concentrations of infectious and surface-fouling bacteria that are indigenous to ocean waters. Both macroalgae (seaweeds) and microalgae (diatoms) contain pharmacologically active compounds such as phlorotannins, fatty acids, polysaccharides, peptides, and terpenes which combat bacterial invasion. The resistance of pathogenic bacteria to existing antibiotics has become a global epidemic. Marine algae derivatives have shown promise as candidates in novel, antibacterial drug discovery. The efficacy of these compounds, their mechanism of action, applications as antibiotics, disinfectants, and inhibitors of foodborne pathogenic and spoilage bacteria are reviewed in this article.

## 1. Introduction

The search for bioactive compounds from marine organisms in recent decades has produced an abundance of extracts with pharmaceutical and industrial applications. In 2013 alone, over one thousand pharmacologically active compounds of marine origin were characterised worldwide, with potential efficacy against cancer, viruses, bacteria, fungi, hypertension, high cholesterol and other diseases. Marine organisms commonly targeted for screening include macro and microalgae, cnidarians, phytoplankton, molluscs, sponges, corals, tunicates, and bryozoans. Much of this research has concerned the identification of antimicrobial agents; particularly compounds active against pathogenic bacteria [1,2]. The global epidemic of bacterial resistance to existing antibiotics such as β-lactams and quinolones has prompted the search for naturally occurring candidate drugs, anti-fouling agents, and food preservation agents from terrestrial and marine sources. The evolution of antibacterial biomolecules alongside bacteria over millions of years may provide them with the potential to overcome strains such as methicillin resistant *Staphylococcus aureus* (MRSA) and fluoroquinolone-resistant *Pseudomonas* [3]. Antibacterials from the marine environment in particular have been studied. Micro and macroalgae, such as diatoms and seaweeds, have developed indigenous systems to combat pathogenic bacteria and other microbes, ubiquitous to the ocean environment. This review focuses on antibacterial compounds derived from marine algae, their mechanism of pharmacological action, and outlines their current and potential applications as antibiotics, disinfectants, and inhibitors of foodborne pathogenic and spoilage bacteria.

## 2. Antibacterial Compounds in Marine Algae and Their Functional Groups

Algae have traditionally been used as food, or for hydrocolloids such as alginate and carrageenan. However, modern screening methods have identified antibacterial compounds in the secondary metabolites of algal classes such as the Phaeophyceae (brown), Rhodophyceae (red), Chlorophyceae (green), Chrysophyceae (golden) and Bacillariophyceae (diatoms) [4,5,6,7,8]. Functional groups with antibacterial activity in these compounds include phlorotannins, fatty acids, polysaccharides, peptides, terpenes, polyacetylenes, sterols, indole alkaloids, aromatic organic acids, shikimic acid, polyketides, hydroquinones, alcohols, aldehydes, ketones, and halogenated furanones, alkanes, and alkenes [9,10,11]. The ecological function of some of these secondary metabolites in algae is not yet fully understood. Since they are not required for normal growth or reproduction, adaption to conditions to which they are exposed in the ocean is proposed to be the main factor controlling their occurrence. Impediments to algal survival include grazing by herbivores, competition for space from other organisms, thallus injury, and biofilm formation. Algae are also exposed to osmotic stress; and high levels of UV light, oxygen, and salinity. In addition, an average of one million bacterial cells are present in each millilitre of seawater [12]. Some of the chemicals produced by algae to combat these stresses have antibacterial potential, and many have high biological activity due to the diluting effect of seawater and the harsh environment in which they live [13].

### Algal Allelopathy

The biological phenomenon of allelopathy in terrestrial and marine habitats contributes to the production of potentially antibacterial compounds. Allelochemicals are released by many algae into their environment. These secondary metabolites exert beneficial or detrimental effects on competing organisms, the end result of which offers a competitive advantage to the alga. Sessile algae such as seaweeds are more prone to bacterial biofilm formation and consumption by invertebrates compared to motile, single cell algae. Allelochemicals have been identified in several species which aid algae in competition for anchoring space on rocks and the seabed. Many of these compounds are toxic, with applications in cancer chemotherapy, and are currently being screened for antibacterial potential [14].

Vieira *et al.* [15] characterised three new lobophorenols (C_21_ polyunsaturated alcohols) in the brown seaweed genus, *Lobophora*, which had significant allelopathic effects against the coral, *Acropora muricata*, causing bleaching and necrosis. Similarly, two acetylated diterpenes reported by Rasher *et al.* [16] in the green alga, *Chlorodesmis fastigiata*, and two loliolide derivatives from the red alga, *Galaxaura filamentosa*, acted as potent allelochemicals against corals. The lipophilic compounds were secreted from thallus surfaces and inhibited photosynthesis in the corals, causing bleaching, and in some cases, death. A kainic acid analogue, domoic acid, produced by the diatom genus, *Pseudo-nitzschia*, acts as an intra and inter-species allelochemical, and is responsible for toxic algal blooms. Domoic acid is an excitotoxic amino acid that affects the central nervous system in vertebrates such as shrimp, and competitively inhibits the growth of other *Pseudo-nitzschia* species [17,18]. Similarly, ovatoxins and palytoxins produced by the toxic dinoflagellate protozoan, *Ostreopsis*, [19] act as allelochemical deterrents to neighbouring vertebrates, and can cause algal blooms. Accoroni *et al.* [20] examined the allelopathic interactions between *Ostreopsis cf.* *ovata* and three seaweeds, *Dictyota dichotoma*, *Rhodymenia pseudopalmata*, and *Ulva rigida* that compete for space in the same habitat. Fresh and dried thalli of *Dictyota dichotoma* and *Rhodymenia pseudopalmata* were found to significantly inhibit the growth of *Ostreopsis ovata*. The compounds responsible are currently being elucidated and have potential applications in the prevention of algal blooms, and as antibacterials.

Phlorotannins and terpenes in algae also act as allelochemical deterrents to herbivores, inhibitors of bacterial biofilm formation, and as antioxidants against UV damage [21,22,23]. Phlorotannins are composed of polyphenolic phloroglucinol units and occur in Phaeophyceae. They have the ability to bind with and precipitate proteins in the same way tannins do in terrestrial plants. This makes the algae more astringent and less palatable to grazers as it binds with proteins in the mouth [24,25,26].

Some seaweeds, such as the green alga, *Caulerpa cylindracea*, have developed immunity to several pathogenic epiphytic *Vibrio* species that live on their thallus surface. A symbiotic allelopathic relationship with the bacteria has been proposed by Rizzo *et al.* [7], where the presence of the *Vibrio* actually contributes to successful algal reproduction, allowing the two organisms to function as a holobiont [27]. Foodborne illness caused by *Vibrio* and other pathogenic bacteria is an increasing global issue. Isolation of the compounds and the mechanism responsible for algal immunity to these bacteria could produce useful therapeutics and sanitisers. The harnessing and bioengineering of recently characterised allelochemicals represents a potential area of new marine antibacterials.

## 3. Mechanisms of Pharmacological Action and Potential Applications

Bactericidal and bacteriostatic compounds were first isolated from algae when chloroform and benzene fatty acid extracts of chlorellin, from *Chlorella vulgaris*, were found to inhibit *Pseudomonas aeruginosa*, *Staphylococcus aureus*, *Streptococcus pyogenes*, and *Bacillus subtilis* [28]. Chlorellin was not suitable for large scale commercial use, however it heralded further research into algal antimicrobial inhibitors in genera such as *Scenedesmus* [29]. As discussed, several chemical functional groups in algae, such as phlorotannins, fatty acids, peptides, terpenes, polysaccharides, polyacetylenes, sterols, indole alkaloids, aromatic organic acids, shikimic acid, polyketides, hydroquinones, alcohols, aldehydes, ketones, and halogenated furanones have been reported as bacterial inhibitors. The mechanism of pharmacological action for some remains uncertain, however, methods of bacterial inhibition employed by the following functional groups have been proposed.

### 3.1. Phlorotannins

Phlorotannins occur in fucosan granules called physodes within algal cells and comprise 1%–15% of the thallus dry mass [30]. The antibacterial activity of phlorotannins has been reported to be due to inhibition of oxidative phosphorylation, and their ability to bind with bacterial proteins such as enzymes and cell membranes, causing cell lysis. The phenolic aromatic rings and OH groups of the phloroglucinol units bind to the -NH groups of bacterial proteins by H-bond and hydrophobic interactions [31,32]. Kamei and Isnansetyo [33] reported that the bacteriolytic activity of phloroglucinol compounds against *Vibrio* species increased when tertiary structures such as methyl- or acetyl-vinyl were present. However, a greater minimum inhibitory concentration (MIC) was required to penetrate the Gram-negative species, *Vibrio* *parahaemolyticus*, compared to the Gram-positive MRSA. This is the case for most antibacterials, due to physiological differences in β-lactamase mechanisms, and the less penetrable nature of the outer lipopolysaccharide membrane of Gram-negative species in comparison to the peptidoglycan Gram-positive layer [34].

Wei *et al.* [35] reported that low molecular weight phlorotannins extracted from *Sargassum thunbergii* damaged the cell membrane and cell wall of *Vibrio* *parahaemolyticus*, causing cytoplasm leakage and deconstruction of membrane permeability. The study suggested that low molecular weight phlorotannins from algae could potentially be used in food safety control and aquacultural drugs. Lee *et al.* [36] tested a range of solvent extracts from the brown seaweed, Arame (*Eisenia bicyclis*) against antibiotic resistant *Propionibacterium*-related acne. A phlorofucofuroeckol compound (phlorotannin with an alcohol substituent) exhibited the most potent antibacterial activity with an MIC of 32 μg/mL, while also significantly reversing the resistance of *Propionibacterium* to erythromycin and lincomycin. The same research group tested the activity of phlorofucofuroeckol from *Eisenia bicyclis* against MRSA. Phlorofucofuroeckol suppressed mecI, mecR1, and mecA gene expression in the resistant *Staphylococcus aureus* cells. These three genes regulate the expression of methicillin resistance in bacteria. This resulted in suppression of penicillin-binding protein 2a production, which is considered the main mechanism by which MRSA strains resist methicillin [37,38]. Phlorotannins and their derivatives offer a potentially useful source of natural antibacterial agents for food and medical applications.

### 3.2. Fatty Acids

Algal free fatty acids have been reported to act as inhibitors of the electron transport chain and normal oxidative phosphorylation in bacterial cell membranes. This interferes with adenosine triphosphate energy transfer, and inhibits enzymes such as bacterial enoyl-acyl carrier protein reductase, necessary for the synthesis of fatty acids within the bacterial cell. Lysis of the cell, and formation of peroxidation and auto-oxidation degradation products then occurs [39,40,41,42]. El Shafay *et al.* [43] identified the antibacterial fatty acids, cyclopentaneacetic acid, and 10,13-octadeadienoic acid as principal components of ethanol extracted *Sargassum vulgare* and diethyl ether extracted *Sargassum fusiforme*. Transmission electron microscopy was used to measure the morphological changes in *Staphylococcus aureus* and *Klebsiella pneumoniae* cells treated with these brown seaweed extracts. The cell walls of both bacteria were perforated, resulting in rupture of the cell wall, cytoplasmic leakage, shrinking of the protoplasm, cytoplasmic vacuolation, scattering of chromatin, distortion of the outer cell shape, and decreased cell size. Čermák *et al.* [44] reported the antibacterial long-chain fatty acids in the green microalga *Planktochlorella nurekis* to be significant inhibitors of *Campylobacter jejuni*, *Escherichia coli*, *Salmonella enterica* var. *Enteritidis*, *Salmonella enterica* var. *Infantis*, *Arcobacter butzleri*, and *Lactobacillus johnsonii* using a suspension concentration range of 0.75–6 mg/mL. The study proposed that green microalgae could be used as an alternative to in-feed antibiotics to prevent disease in livestock and poultry and to maintain the microbial safety of animal products in the human food chain.

### 3.3. Polysaccharides

Polysaccharides are composed of repeating monosaccharide units linked by glycosidic bonds. They function primarily as structural storage compounds in plants and algae. Algal polysaccharides and sulphated polysaccharides have been used successfully for pharmaceutical and dietary applications. Their mechanism of antibacterial action is proposed to be due to glycoprotein-receptors present on the cell-surface of polysaccharides which bind with compounds in the bacterial cell wall, cytoplasmic membrane, and DNA. This results in increased permeability of the cytoplasmic membrane, protein leakage, and binding of bacterial DNA [45,46,47]. Polysaccharides, such as fucoidan and laminarin, have been successfully used in drug delivery as oral antibiotics to inhibit the growth of *Staphylococcus aureus* and *Escherichia coli*; and to prevent the adhesion of *Helicobacter pylori* biofilms in gastric mucosa. They have also been incorporated into food as a dietary supplement to improve the immune response of farmed fish, and reduce susceptibility to *Piscirickettsia salmonis* infection [48,49,50,51].

Kadam *et al.* [48] reported a significant inhibition of *Staphylococcus aureus*, *Listeria* *monocytogenes*, *Escherichia coli* and *Salmonella* *typhimurium* growth with ultrasound assisted extraction of laminarin from the Irish brown seaweeds *Ascophyllum nodosum* and *Laminaria hyperborea* using 0.1 M hydrochloric acid. Abou Zeid *et al.* [52] demonstrated that hot and cold water-extracted polysaccharides from the red seaweed *Pterocladia capillacea* and brown seaweed *Dictyopteris membranacea* inhibit the growth of Gram-positive *Bacillus* *cereus* and *Staphylococcus aureus*, and Gram-negative *Pseudomonas fluorescens* and *Escherichia coli* in disc diffusion assays. In the case of *Staphylococcus aureus*, cold water-extracted *Pterocladia capillacea* had an activity equivalent to 56.8% of the antibiotic standard, ampicillin. Vijayabaskar *et al.* [53] found that sulphated polysaccharides extracted from *Sargassum swartzii* inhibited both Gram-positive and Gram-negative bacteria in ten human pathogenic strains. In the case of *Escherichia coli*, the polysaccharide extract was more potent in the disc diffusion assay than an ampicillin antibiotic standard.

### 3.4. Proteins and Peptides

The antimicrobial activity of amino acids in the form of short-chain peptides, or larger, more complex proteins, has been demonstrated in a number of recent studies. The amphipathic conformation of peptides enables them to bind with polar and non-polar sites on bacterial cytoplasmic membranes, thereby interfering with cellular processes and propagation [54,55,56].

Lectins, for example, are a diverse group of proteins that occur in animals, plants, algae, bacteria, and viruses [57]. In humans, they have multiple biological functions including carbohydrate-binding, cell adhesion, blood-protein regulation, and immune defence [58]. The ability of lectins to selectively bind with lipopolysaccharides, β-glucans, and peptidoglycans on the cell surface of bacteria affords them bactericidal properties, as normal cell processes such as nutrient uptake are blocked [59]. Several functional groups are involved. Hydrogen bonds are formed between polar moieties of amino acids in the lectins and the hydroxyl groups of polysaccharides, coupled with van der Waals interactions, and packing of hydrophobic polysaccharide regions against amino acid aromatic groups in the lectins [60,61,62]. Holanda *et al.* [63] evaluated the inhibitory effect of lectin extracts from the red alga *Solieria filiformis* against Gram-negative and Gram-positive pathogenic bacteria. At a concentration of 1000 µg/mL, the extract inhibited growth of the Gram-negative species *Pseudomonas aeruginosa*, *Enterobacter aerogenes*, *Serratia marcescens*, *Salmonella typhi*, *Klebsiella* *pneumoniae*, and *Proteus* species. The binding of lectin with mannan was believed to be the method by which growth was inhibited. Mannan is a linear polymer of the saccharide monomer mannose, and occurs on the cell surface of Gram-negative bacteria. Mannan acts as a hapten upon binding with the large lectin molecule, eliciting an immune response. However, no inhibition of growth was observed against the Gram-positive *Staphylococcus aureus* and *Bacillus* *subtilis*, possibly due to unsuitable lectin-polysaccharide binding sites on the cell surfaces of these species [64].

Enzymatic hydrolysis is the most common method used to isolate bioactive peptides from parent proteins for medical applications, thus avoiding toxic solvent residues. The bioactivity of individual peptide hydrolysates is often distinct from, or greater than that of the original protein. Some recently characterised peptides have been termed crypteins, due to their cryptic, or hidden, bioactivity, and are a current area of novel therapeutic study [65]. Marine algae are good candidates for the mining of such peptides as they contain a high proportion of diverse proteins [66,67].

Beaulieu *et al.* [68] extracted antibacterial peptides (>10 kDa mass) from the brown seaweed *Saccharina longicruris* by enzymatic hydrolysis with trypsin. Liquid chromatography-tandem mass spectrometry identified the sub-fractions as peptide precursors to proteins similar to ubiquitin, leucine, histone, and a ribosomal structure, which form part of the innate immune defence of the seaweed. Maximum specific growth rate of the food spoilage bacterium *Staphylococcus aureus* was significantly inhibited by the hydrolysate at concentrations of 0.31 mg/mL to 2.5 mg/mL making it a potential agent for food preservation.

### 3.5. Terpenes

Terpenes are compounds composed of repeating isoprene units, often with substituent groups. Terpenes with pharmacological activity have been successfully extracted from terrestrial plants. For example, paclitaxel (or taxol) from Pacific yew trees is used in cancer treatment, and Artemisinin from the plant *Artemisia annua* is an anti-malarial drug [69,70]. A number of terpene compounds from algae, such as diterpene-benzoate bromophycolides, have been found to inhibit bacterial growth. Lane *et al.* [71] extracted bromophycolides (diterpene-benzoate macrolides) from the Fijian red alga *Callophycus serratus* with water, methanol, and dichloromethane. Extracts significantly inhibited MRSA and vancomycin-resistant *Enterococcus faecium*, with maximal inhibitory concentration (IC_50_) values of 1.4 µM and 5.8 µM respectively. Their findings suggested that the mechanism of antibacterial action was due to the hydrophobicity and conformational rigidity of the tetrahydropyran structure. Rodrigues *et al.* [72] used dichloromethane to isolate sphaerane bromoditerpenes, including a previously uncharacterised, rare dactylomelane called sphaerodactylomelol, from the red alga *Sphaerococcus coronopifolius*. The extracts were found to inhibit *Escherichia coli*, *Pseudomonas* *aeruginosa*, *Staphylococcus aureus*, and *Candida* *albicans*. The greatest antibacterial was observed against *Staphylococcus aureus*, with an IC_50_ value of 6.35 µM. Etahiri *et al.* [73] isolated bromosphaerone and 12*S*-hydroxybromosphaerodiol from the same alga, *Sphaerococcus coronopifolius*. Bromosphaerone and 12*S*-hydroxybromosphaerodiol inhibited *Staphylococcus aureus* with MIC values of 0.104 μg/mL and 0.146 μg/mL respectively. It was proposed that the antibacterial activity of bromosphaerone was due to its amphipathic structure of three polar alcohol groups, with non-polar aliphatic carbon and bromine atoms on the opposite side of the molecule, enabling it to bind with bacterial cell membranes [13,73].

Xanthophylls, such as lutein and astaxanthin, are tetraterpene oxygen-containing compounds that function as light harvesting pigments in plants and algae. They have been extensively documented for their antioxidant activity, but are also effective against bacteria. Fucoxanthin is a xanthophyll that occurs predominantly in brown algae (Phaeophyceae), diatoms (Bacillariophyceae), and at lower concentrations in golden algae (Chrysophyceae) and Raphidophyceae [74,75,76,77,78]. Rajauria and Abu-Ghannam [79] extracted fucoxanthin from the Irish brown seaweed *Himanthalia* *elongata* using diethyl ether, n-hexane, and chloroform, and purified the crude extract using preparative TLC. In disc diffusion assays, the purified extract was shown to be a potent inhibitor of *Listeria* *monocytogenes*, with an inhibition zone of 10.27 mm at a concentration of 1 mg/mL (25 μg/disc). The extract was 98.4% as effective as an analytical grade fucoxanthin standard (inhibition zone 10.89 mm). Similarly, Deyab and Abou-Dobara [80] extracted fucoxanthin from the brown seaweed *Turbinaria triquetra*, the green *Ulva lactuca*, and the red *Laurencia obtusa* with chloroform and methanol. Extracts were purified by silica column chromatography and identified by nuclear magnetic resonance spectroscopy. *Turbinaria triquetra* showed the greatest bacterial inhibition, followed by *Laurencia obtusa*. *Ulva lactuca* extracts had significantly lower antibacterial activity. Zones of inhibition for *Escherichia coli*, *Bacillus cereus*, *Bacillus subtilis*, *Klebsiella pneumoniae*, *Staphylococcus aureus*, and *Pseudomonas aeruginosa* ranged from 4.0 mm to 7.0 mm (100 µg/mL extract/disc) and in the case of some Gram-negative species, were equivalent to antibiotic standards.

### 3.6. Chrysophaentins

Plaza *et al.* [81] isolated eight compounds from the chrysophyte alga *Chrysophaeum taylori* using hexane, chloroform, and methanol. The compounds were of a new chemical structural class which they named chrysophaentins, which consisted of two polyhydroxylated, polyhalogenated ω,ω′-diarylbutene units connected via two ether bonds. The chrysophaentins exerted powerful inhibition *in vitro* of MRSA (MIC = 1.5 μg/mL), vancomycin-resistant *Enterococcus* *faecium* (MIC = 2.9 μg/mL), and multidrug-resistant *Staphylococcus aureus* (MIC = 1.3 μg/mL). The pharmacological mechanism of action of chrysophaentin is proposed to be unlike any existing antibacterial agent. The functional groups within chrysophaentin act as enzyme inhibitors by binding with guanosine triphosphatase in bacterial cells. This prevents the synthesis of a protein called FtsZ (filamenting temperature-sensitive mutant Z), required for bacterial cell division. The development of antibiotics and other antibacterial products from chrysophaentin continues to be an important area of marine pharmacological investigation [82,83].

### 3.7. Lactones

Lactones are a chemical class of cyclic esters, which includes furanones. The Australian red seaweed *Delisea pulchra* has been studied for its ability to remain free of surface bacterial colonisation. Halogenated furanone extracts from *Delisea pulchra* have been used as effective surface sanitisers in the prevention of *Pseudomonas aeruginosa* biofilm formation. This halogenated furanone also inhibits quorum sensing mechanisms by interfering with bacterial inter-cell communication. In order for bacteria to express specific genes during quorum sensing, signalling molecules called acyl-homoserine lactones (AHLs) are required, as well as luminescence transcriptional activator (LuxR) regulatory proteins. The furanone extract from *Delisea pulchra* competes with AHL for the LuxR receptor site, thereby inhibiting virulence factor production and pathogenesis in *Pseudomonas aeruginosa* [84,85]. Ren *et al.* [86] found a similar inhibition of quorum sensing in *Escherichia coli* with a (5*Z*)-4-bromo-5-(bromomethylene)-3-butyl-2(5*H*)-furanone extract from *Delisea pulchra*. Quorum sensing in *Escherichia coli* was inhibited by blocking *S*-ribosylhomocysteine lyase (LuxS) mediated AI-2 signalling. This influences genes and proteins involved in the normal production of flagellar synthesis, motility, and chemotaxis in the bacterium. Manefield *et al.* [87] identified another mechanism of inhibition exerted by a halogenated furanone from *Delisea pulchra*. The bacterium, *Erwinia carotovora*, produces carbapenem as a virulence factor during quorum sensing. A commercially available 4-bromo-5-(bromomethylene)-3-(1′-hydroxybutyl)-2(5*H*)-furanone was found to inhibit carbapenem production in *Erwinia carotovora* by disrupting the 3-oxo-*C*6-HSL dependent expression of the *carABCDEFGH* operon. Castillo *et al.* [88] also reported that a commercially produced furanone, similar to the *Delisea pulchra* extract, was effective against Gram-negative *Campylobacter jejuni*. When combined with epigallocatechin gallate from green tea and a citric acid extract, AI-2 activity, bacterial motility, and biofilm formation was significantly decreased.

Considering the efficacy of the *Delisea pulchra* extract against the Gram-negative *Pseudomonas* *aeruginosa* and *Escherichia coli in vitro*, it is not unreasonable to propose that it could also inhibit Gram-negative *Campylobacter jejuni.* Algal furanones and other lactones may have potential as alternatives to synthetic surface sanitisers, and antibiotics. *Campylobacter jejuni* and *Escherichia coli* are two of the leading causes of food poisoning worldwide, and have developed resistance to many traditional antibiotics [89]. The demonstrated ability of algal furanone extracts to prevent biofilm formation may be useful in the treatment of *Pseudomonas* *aeruginosa* infection, which is characterised by the formation of a mucoid film in the lungs of cystic fibrosis sufferers. *Pseudomonas* *aeruginosa* is also an increasing risk to the immunocompromised, such as premature babies, and long-term hospital patients [90].

Algal polysaccharides, fatty acids, peptides, proteins, phlorotannins, terpenes, chrysophaentins, and lactones make good candidates for antibiotics, and incorporation into human food products for safety and preservation as they are edible, non-toxic, and inexpensive. Further research, including *in vivo* toxicology studies, into these antibacterial extracts could produce very useful food preservation and pharmaceutical products.

## 4. Effect of Extraction Methodology on Antibacterial Potency

### 4.1. Environmental Influences

A number of factors influence the efficacy of algal antibacterial compounds and should be taken into consideration when comparing extraction methods. Natural factors such as ontogenetic and environmental influences, seasonal variations, ocean temperature, salinity, and pollutants have a profound influence on the content and potency of algal bioactives. For example, the alginate, mannitol, laminarin, and polyphenolic contents of *Laminaria* *digitata, Laminaria* *hyperborea, Saccharina latissima*, and *Alaria esculenta* are known to vary significantly throughout the year. In *Laminaria* species sampled off the west Scottish coast, polyphenols have been reported to accumulate with the on-set of the growth phase between May and July and reach their lowest levels in October [91,92]. Bioactive compounds are present at fluctuating concentrations during different stages of the algal life cycle. In addition, distribution varies in each discrete part of the thallus, *i.e.*, holdfast, blade, and stipe [93]. Sampling of each section of the thallus, and analysis of the same species at different points throughout the year provides more accuracy.

### 4.2. Extraction Method and Solvent

The means by which algal antibacterial compounds are extracted from raw biomass impacts their pharmacological efficacy. The extraction method and solvent choice influence the physical and chemical properties of the extract. Various extraction protocols for the recovery of antibacterials from marine algae have been reported. Some involve freeze drying, Soxhlet extraction, and the use of organic solvents, enzymes, or bacterial fermentation. Maceration, often with liquid nitrogen, is generally required for algae, in order to release cell components from the tough, polysaccharide-rich thallus of seaweeds, and the silica-bound cell walls of microalgal diatoms. This exposes them to the extraction solvent. Protocols employ different extraction temperatures, times, pH ranges, and concentrations. All these variables influence the efficacy of the final antibacterial product.

#### 4.2.1. Solvent Polarity

The polarity of the extraction solvent profoundly affects the manner in which the extract will interact with functional groups on bacterial surfaces. For example, depending on the polarity of the solvent used, functional groups extracted may be polar hydrophilic or non-polar lipophilic. This influences the ability of the algal extract to act upon the cell membrane and other components of Gram-positive and Gram-negative bacteria. In a recent study, Moorthi and Balasubramanian [94] compared acetone, methanol, and chloroform Soxhlet extraction in the evaluation of the antibacterial efficacy of the brown seaweed *Sargassum muticum* harvested in India. Disc diffusion assays were used to measure its efficacy against foodborne human pathogenic bacteria *Salmonella paratyphi*, *Staphylococcus epidermis*, *Enterobacter aerogenus*, *Klebsiella pneumoniae*, *Shigella fleschneri*, *Proteus vulgaris*, MRSA, and *Salmonella typhimurium*. At concentrations of 30 µL/mL, acetone extracts were significantly more potent than chloroform or methanol extracts against *Salmonella paratyphi*, *Salmonella typhimurium*, *Staphylococcus aureus* and *Shigella* *fleschneri*. Chemical analysis of the *Sargassum muticum* acetone extracts showed the presence of tannins. The effectiveness of acetone for the extraction of antibacterials from *Sargassum wightii* and *Caulerpa scalpelliformis* has previously been reported, and attributed to the ability of polar acetone to bind with the hydrophilic phlorotannins in seaweed which, as discussed, have potent antibacterial activity [95,96].

Since lipophilic compounds in algae also have antibacterial activity, non-polar solvents such as hexane, or supercritical carbon dioxide are also commonly employed. Mendiola *et al.* [97] used supercritical CO_2_ for 60 min, at 40 °C, 400 atm to extract lipids from the microalga *Chaetoceros* *muelleri*. The free fatty acids extracted significantly inhibited *Escherichia coli* and *Staphylococcus aureus* growth. Unlike most organic solvents, supercritical CO_2_ has the benefit of being environmentally friendly since it is gaseous at room temperature and pressure, leaving no trace in the extracts. Solvent-free antibacterial extracts such as these have potential as food preservation ingredients.

Extraction yield is also impacted by solvent choice, which in turn affects efficacy of the antibacterial. Rajauria *et al.* [98] reported different combinations of methanol and water (20%–80%) to have a significant effect on the yield of antimicrobial and antioxidant polyphenolic compounds from the Irish brown seaweed *Himanthalia elongata*. The greatest yield (6.8%) was achieved using 60% methanol, compared to the lowest yield (1.2%) using 100% methanol. Disc diffusion and broth dilution tests showed the 60% methanol extract at a concentration of 60 mg/mL to be the most potent inhibitor of Gram-positive *Listeria monocytogenes* and *Enterococcus faecalis*; and Gram-negative *Pseudomonas aeruginosa* and *Salmonella abony*. The *Himanthalia elongata* extract had greater, or at least equal, zones of inhibition against all bacteria compared to the synthetic food preservatives sodium nitrite and sodium benzoate. Cox *et al.* [5] also found that the antibacterial activity of polyphenolic seaweed extracts was solvent-dependent. Methanol was determined to be the most effective for the brown seaweeds *Himanthalia elongata*, *Laminaria* *digitata*, and *Saccharina latissima*; while acetone and ethanol were more effective for the green species *Enteromorpha spirulina*; and the red species *Palmaria* *palmata* and *Chondrus* *crispus*. Antibacterial activity was tested on food spoilage (*Enterococcus faecalis* and *Pseudomonas aeruginosa*) and pathogenic (*Listeria monocytogenes* and *Salmonella abony*) bacteria using the microwell plate method. Using methanol only, green and red seaweed extracts had significantly lower antibacterial activity than the brown species, however their potency increased significantly when ethanol and acetone were used as solvents. In the case of *Chondrus* *crispus*, *Enterococcus faecalis* inhibition increased from 39% to 100% using ethanol and acetone. Methanol extracts of *Himanthalia elongata* showed 100% inhibition of *Listeria monocytogenes*, *Enterococcus faecalis*, and *Salmonella abony*, which was, on average, 4.34% greater than that of sodium nitrite and sodium benzoate. *Laminaria* *digitata* also inhibited *Listeria monocytogenes* by 100%.

Since the structure and solubility of algal antibacterial compounds varies so greatly, using a series of solvents with an incremental range of polarities and concentrations would ensure the extraction of polar and non-polar fractions.

#### 4.2.2. Food Grade Solvents

Solvent choice also influences potential applications of algal antibacterial extracts in terms of human and animal consumption. Many organic solvents such as methanol and dimethyl sulphoxide (DMSO) produce high extraction yields, but leave toxic levels of residue. A number of food-grade solvents have been approved for use in the extraction of bioactives for consumption. Since 1966, The Food Chemicals Codex has been recognised internationally as the authority for agreed standards in ingredients, preservatives, extraction solvents, enzymes, and bacteria that are permissible for food use [99]. The European Union also issues Directives on food-approved solvent systems and residual limits, which are administered by the European Food Safety Authority. For example, Commission Directive 2010/67/EU sets out the maximum acceptable residues for acetone, hexane, and ethanolic extracts of rosemary. The acceptable concentrations are given as not more than 500 mg/kg acetone; 25 mg/kg hexane; and 500 mg/kg ethanol. The Directive also recommends further purification of the extract with active carbon and/or molecular distillation [100]. Alternatively, solvents that are generally recognised as safe (GRAS) under Sections 201 and 409 of the U.S. Federal Food, Drug, and Cosmetic Act can be utilised, with no issues of toxicity [101]. For example, in a recent study, Boisvert *et al.* [102] used pressurised liquid extraction with approved, food-grade ethanol to extract high yields of antibacterial and antioxidant-rich compounds from three species of seaweed, *Ascophyllum* *nodosum*, *Ulva lactuca*, and *Saccharina longicruris*, from the St Lawrence Estuary, Canada. Antibacterial activity was tested against three food spoilage bacteria, *Micrococcus luteus*, *Escherichia coli*, and *Brochothrix thermosphacta*, using the microtiter method. The greatest inhibition of *Escherichia coli* (69.5%), *Micrococcus luteus* (61.4%), and *Brochothrix* *thermosphacta* (21.4%) was exerted by *Ulva lactuca* extracts at a concentration of 500 μg/mL. The greater potency of the *Ulva* *lactuca* compared to the other species was thought to be due to a higher mineral concentration of metals such as copper, zinc, silver and mercury, which have innate antibacterial activity. This choice of food grade solvent and green extraction method exemplifies some of the approaches that can be used in the development of safe algal antibacterials.

### 4.3. Novel Extraction Technologies

Aside from traditional solid-liquid extraction, a number of novel technologies have been developed with the aim of improving yields, minimising damage to analytes, reducing solvent waste, and conserving energy and time. These can have significant effects on the yield and potency of algal antibacterial extracts. Technologies include extraction assisted by microwaves, pulsed electric fields, pressurised solvents, supercritical fluids, ultrasound, and enzymes [103]. The latter three technologies are most applicable to algal extraction, and are outlined here.

#### 4.3.1. Supercritical Fluid Extraction

When brought to a pressure and temperature above its critical point, a fluid or gas becomes supercritical. While in this state, it possesses the physical properties of a gas and can effuse through solids; but can also act as a liquid solvent [104]. These properties are highly useful for the extraction of bioactive compounds from organic tissues. Carbon dioxide is most commonly used for food and pharmaceutical applications as it completely dissipates after extraction at room temperature. Coffee, for example, is decaffeinated using supercritical CO_2_. As discussed in Section 4.2.1, Mendiola *et al.* [97] used CO_2_ supercritical fluid extraction to isolate lipids with antibacterial activity from the microalga *Chaetoceros* *muelleri*. A highly significant increase in antibacterial activity was reported using this method in comparison to traditional methodologies. To compare the efficiency of their method, a liquid-liquid DMSO flask extraction of raw *Chaetoceros* *muelleri* was prepared. A broth microdilution method was used to evaluate the MIC value of each extract against *Escherichia* *coli* and *Staphylococcus aureus*. The supercritical fluid extractions exerted three times more bacterial inhibition than the DMSO extract. Despite the increased energy demands of supercritical fluid extraction, it offers a green chemical alternative to solvent based methods for antibacterial agents destined for food and pharmaceutical uses.

#### 4.3.2. Ultrasound Assisted Extraction

Ultrasound or ultrasonic technology uses high intensity sound waves to penetrate a liquid, producing alternating high and low pressure cycles. During low-pressure, high-intensity ultrasonic waves create tiny vacuum bubbles. When the high-pressure cycle begins they implode, or cavitate, releasing liquid jets at extremely high velocity. This high shear force ruptures the cells of organic tissue, releasing their contents [105]. This has a positive effect on the yield and potency of bioactive extracts. Kadam *et al.* [48] compared the efficiency of ultrasound assisted and solid-liquid flask extraction of laminarin from the Irish brown seaweeds *Laminaria* *hyperborea* and *Ascophyllum nodosum*. Two solvents were compared for both extraction methods; water, and 0.1 M hydrochloric acid. As discussed, laminarin is an algal polysaccharide with antibacterial activity. The percentage yield of laminarin was significantly greater in both water and HCl ultrasound assisted extracts for both seaweeds. *Laminaria hyperborea* ultrasound/HCl yield was 6.240%, compared to only 3.254% from the flask/HCl extraction. The ultrasound assisted yield was 47.85% greater. Similarly, *Ascophyllum nodosum* ultrasound/HCl yield was 5.822%, compared to 4.304% from the flask/HCl extraction. The ultrasound assisted yield was 26.08% greater. The ultrasound assisted water extracts of *Laminaria* *hyperborea* (5.975%) and *Ascophyllum nodosum* (5.290%) were also greater than the water flask extractions (4.362% and 4.599% respectively). Aside from the substantial increase in laminarin yields, the ultrasound process was also far more energy efficient, requiring only 15 min in a 750 W ultrasonic processor, at an amplitude level of 60%; compared to 2.5 h at 70 °C for solid-liquid extraction. Ultrasound assisted extraction is a suitable technology for deriving antibacterial compounds from algae, as it obviates the need for high temperatures, toxic solvents, and prolonged extraction times. It also fractionates the hardy, complex cell walls of algae [106].

#### 4.3.3. Enzyme Assisted Extraction

Extraction through the application of enzymes, such as cellulases, has the potential to increase the yield and safety of algal antibacterials. The presence of branched, sulphated, or complex polysaccharides, such as alginate and laminarin, in algal cell walls limits the efficiency of classical extraction methods [11,107]. Food-grade digestive enzymes, such as proteases and carbohydrases, could aid in the degradation of cell wall matrices, thus releasing cell components. To date, little has been published on the use of enzymes for the extraction of antibacterial compounds from algae specifically. However, in the extraction of algal bioactives such as antioxidants, polysaccharides, carotenoids and polyphenols, the use of enzymes has shown significant potential as a viable alternative, or addition to, pure solvent methods [108,109,110,111]. Since antioxidants, polysaccharides, carotenoids, and polyphenols, have proven antibacterial activity, it is expected that their enzymatic extracts could also be used to inhibit bacteria. For example, there is an implied correlation between antioxidant activity and antibacterial activity, since algal antioxidant compounds, like fucoxanthin, have been shown to be potent antibacterials. Enzyme assisted extraction could potentially increase extraction yields of algal antibacterials. Bioactive yields from terrestrial plants, such as the carotenoid lycopene from tomatoes, have been increased by enzymolysis. Zuorro *et al.* [112] reported an eighteen fold increase in lycopene yield from tomato processing waste in pectinase and cellulase pre-treated samples, compared to hexane extraction alone. In the extraction of fucoxanthin from *Undaria* *pinnatifida*, Billakanti *et al.* [113] reported a 9.3% increase in fucoxanthin yield using an enzyme pre-treatment of alginate lyase, followed by dimethyl ether and ethanol extraction, compared to non-enzyme treated. Optimum parameters for alginate lyase pre-treatment were 37 °C, for 2 h, pH 6.2, 5% (*w*/*v*) solids, with 0.05 wt % enzyme.

Each of these novel extraction technologies has the potential to replace or reduce solvent driven methods for the recovery of algal antibacterial compounds. Initial set-up costs may be more costly than traditional solvents, but there are several long-term advantages. Supercritical fluid extraction, ultrasound assisted extraction, and enzyme-assisted extraction are non-toxic, environmentally harmless, and in many cases, increase product yields.

## 5. Marine *vs.* Terrestrial Antibacterials

Both marine and terrestrial organisms produce secondary metabolites that protect them from bacterial infection. It is serendipitous that these metabolites also have activity against many pathogenic bacteria which affect humans and animals. Marine algal extracts, such as those discussed in this article, along with terrestrial plant extracts like lavender and tea tree oil, have been used throughout history as natural antimicrobials and as medicines in pharmacognosy. Since marine and terrestrial organisms face vastly different environmental challenges, the structural features and pharmacological activity of their metabolites differ greatly [114]. It has been proposed that some marine antibacterial compounds, from organisms such as algae, have greater antibacterial efficacy than those from terrestrial sources. This has been attributed to the greater bacterial cell numbers in seawater compared to air, and the need for sessile organisms to prevent surface biofouling in the ocean [115].

In study by Wang *et al.* [32] the bacteriostatic and bactericidal activity of methanol extracted phlorotannins from *Ascophyllum nodosum* harvested in Nova Scotia was compared with hydrolysable and condensed tannins from two trees, Quebracho (*Schinopsis* *balansaei*) and Chinese sumac (*Rhus semialata*). Four strains of *Escherichia coli* resistant to ampicillin, kanamycin, and nalidixic acid were used to measure inhibition at OD_600_. At a concentration of only 25 μg/mL, the phlorotannins from *Ascophyllum nodosum* exerted bacteriostatic effects on three of the strains for up to 24 h, after which time two strains resumed growth. Phlorotannins were fully bactericidal to *Escherichia coli* at 50 μg/mL in the case of two strains, and at 100 μg/mL in the case of the other two. The hydrolysable and condensed tannins from the tree extracts were compared at the same concentrations against two of the *Escherichia coli* strains. However, condensed tannins did not exert a bactericidal effect against either of the two strains, and only had bacteriostatic activity against one strain for 6 h. Hydrolysable tannins had no bactericidal or bacteriostatic activity against either of the two strains. The treated and untreated *Escherichia coli* cells were examined by transmission electron microscopy. The membrane structure of the untreated cells was smooth and even. The action of the tannins was apparent on the cell walls of treated samples, particularly in the case of *Ascophyllum nodosum*, where disorganised structures and electron-dense precipitated deposits were visible. The significant differences demonstrated between the marine algal and terrestrial tannins may be attributed to their chemical structure. Although all tannins are composed of cyclic phloroglucinol groups, terrestrial hydrolysable and condensed tannins have a maximum of three rings, whereas algal phlorotannins have up to eight. This means phlorotannins have more hydroxyl groups, enabling them to produce more hydrogen peroxide under aerobic conditions. Hydrogen peroxide is toxic to bacteria [11]. Hydroxyl groups can also form hydrogen bonds with proteins on the bacterial surface, further denaturing the cell.

Although this study by Wang *et al.* [32] clearly illustrates the comparative activity of marine algal *vs.* terrestrial antibacterial compounds, further comparisons and research into this area with a wide range of chemical classes is required.

## 6. Inhibition of Foodborne Pathogenic and Spoilage Bacteria in Food Production

Incidents of food poisoning have increased dramatically in the last few decades due to changes in consumer eating habits, and the growing resistance of spoilage and pathogenic foodborne bacteria to antibacterial agents [116]. With increasing pressure from the public, food manufacturers are moving away from traditionally used synthetic preservatives, and high levels of salt towards naturally derived alternatives. These natural antimicrobials derived from terrestrial plants and marine algae offer shelf-life extension and increased safety from bacteria that cause food poisoning, without the side effects associated with many synthetic preservatives or high salt intake.

Gupta *et al.* [117] evaluated the antibacterial activity of three edible Irish brown seaweeds, *Himanthalia* *elongata*, *Saccharina latissima*, and *Laminaria digitata* in raw and heat processed (95 °C) form. Their activity was tested against pathogens which commonly cause problems in the food industry, *Listeria monocytogenes*, *Salmonella* *abony*, *Enterococcus* *faecalis*, and *Pseudomonas* *aeruginosa*. In microtiter assays, methanol extracts of raw *Himanthalia* *elongata* (60 mg/mL) inhibited *Listeria monocytogenes* by 98.7%, compared to 96.5% inhibition by the synthetic preservative standard sodium benzoate, and sodium nitrite (96.2%). Raw *Himanthalia* *elongata* was also more potent than the standards against *Enterococcus* *faecalis* and *Pseudomonas* *aeruginosa*. Raw *Saccharina latissima* extracts were almost as potent against all four bacteria, followed by *Laminaria digitata*. Heat treatment significantly reduced the antibacterial activity of all seaweeds. These raw seaweed extracts may be useful for incorporation into products such as raw meats and fish, as they would exert their bacterial inhibition during the uncooked, cold storage phase, before inactivation by heat, at which stage the product would be consumed. A recent study by Dussault *et al.* [118] explored the potential of developing several commonly consumed Pacific Island seaweeds into food preservation agents. In broth dilution assays, methanol extracts of the brown species, *Padina* and *Dictyota*, were found to inhibit the growth of Gram-positive foodborne pathogens *Listeria monocytogenes*, *Bacillus cereus*, and *Staphylococcus aureus* at low concentrations (≤500 μg/mL). However, the extracts had no activity against Gram-negative species, possibly due to the inability of the moderate to low polarity, hydrophobic extracts to breach the hydrophilic lipopolysaccharide, Gram-negative bacterial membrane. The extracts are not known to have any toxicity at the concentrations used, making them good candidates for incorporation into foods prone to Gram-positive bacterial growth.

### 6.1. Active Food Packaging Applications

Marine algal compounds also have potential for use as components of antibacterial films made from biodegradable materials which are widely used in active food packaging applications. Several edible food coatings from alginates and carrageenan have been developed [119,120]. Olaimat *et al.* [121] reported a significant reduction in viable *Campylobacter jejuni* numbers on vacuum-packed raw chicken breasts using a carrageenan and chitosan based coating containing heat-treated oriental mustard extract. Although the mustard contained a natural antibacterial compound, allyl isothiocyanate, chicken treated with a mustard-only extract coating did not have significantly lower *Campylobacter jejuni* numbers than the untreated control-coated chicken after 21 days storage. The carrageenan-chitosan-mustard coated samples reduced *Campylobacter jejuni* numbers by up to 2.78 log_10_ CFU/g more than the control coating at 21 days. Lactic acid bacterial growth was also reduced. Methods of controlling *Campylobacter jejuni* would be highly beneficial to poultry based food producers. Gram-negative, microaerophilic *Campylobacter jejuni* is one of the most frequent causes of foodborne illness worldwide, with hens being the main source in most disease cases [122].

Rodríguez-Martínez *et al.* [123] formulated a new, active biodegradable film based on polylactic acid and brown seaweed. An extrusion process was used to incorporate 8% dried *Fucus* *spiralis* seaweed extract and 0.5% natural sorbic acid into a polylactic acid based biodegradable film. The migration values of the film components into food simulants were found to be generally within the maximum acceptable EU limit. It was concluded that the film was suitable for further development as a packaged food protectant and for shelf-life extension. *Fucus* *spiralis* was selected because it showed the greatest inhibition in disc diffusion assays of the foodborne bacteria *Staphylococcus aureus, Bacillus cereus, Bacillus subtilis*, *Pseudomonas fluorescens*, *Escherichia coli*, *Klebsiella pneumoniae*, *Aeromonas hydrophila*, *Vibrio alginolyticus* and *Vibrio parahaemolyticus*, when compared to other seaweed genera, *Ascophyllum, Ulva, Bifurcaria*, and *Gracilaria*. A concentration of only 10 µg/mL of *Fucus* *spiralis* was required to exert zones of inhibition up to 15 mm (for *Bacillus subtilis*). Although sorbic acid has antimicrobial properties against fungi and bacteria, the addition of *Fucus* *spiralis* extract reduces the amount required, and broadens the scope of antibacterial activity. Sorbic acid was first isolated from mountain ash berries, and has antimicrobial action mainly against yeasts and moulds, but is selective against bacteria [124]. It can also be detected as an acrid taste by some consumers. The addition of small amounts of dried seaweeds, or antibacterial extracts of marine algal compounds to active food-films could minimise the volume of traditional preservatives required. This could reduce costs and bring concomitant nutritional benefits.

### 6.2. Animal Feed Supplementation and Aquaculture

Antibacterial algal extracts such as laminarin and fucoidan can also be incorporated into animal feed to improve the safety and nutritional profile of animal products destined for human consumption [9,125,126]. Moroney *et al.* [127] reported an enhancement of pork quality in meat from pigs fed extracts of *Laminaria digitata* containing laminarin and fucoidan for three weeks prior to slaughter. Animals were fed either 450 mg or 900 mg laminarin and fucoidan per kg feed. In both cases, pork meat was found to have an improved fatty acid profile without loss of lipid stability. Saturated fatty acid content was significantly lowered and lipid oxidation was reduced. Aquacultural food products can be a source of foodborne pathogenic and spoilage bacteria. *Vibrio* genera are a common source of foodborne illness in fish products. Seaweeds have been shown to have antibacterial properties against many species that infect farmed fish, which in turn reduces the occurrence of pathogenic bacteria in the final food product [128]. Roohi Fatima *et al.* [129] enhanced the microbial safety of farmed Mozambique tilapia fish by adding an antibacterial methanol extract of the red seaweed, *Portieria hornemannii*, to housing tanks (1 part per trillion per litre). The instances of *Vibrio parahaemolyticus* infection amongst the fish were significantly decreased compared to the untreated group. Histology tests also showed the same significant decrease of *Vibrio* *parahaemolyticus* in muscle tissue.

### 6.3. Consumer Acceptance

Preservatives are necessary to ensure the safety of perishable foods such as meat, fish, and ready to eat products. Consumer trends are influencing the use and development of natural alternatives to synthetic preservatives such as sodium benzoate and sodium nitrite which have associated health side-effects. A number of antibacterial extracts from terrestrial plants have already gained consumer acceptance. Plant extracts with pharmacological activity such as linalool, thymol, and camphor have been used successfully as preservatives in many commercial products [130]. Existing consumer acceptance of preservatives from plants such as citrus fruits, thyme, rosemary, basil, lemon balm, and marjoram may aid the introduction of marine algal antibacterials to mainstream foods. However, organoleptic impacts must be assessed with sensory trials as well as any interactions with food matrices. The antibacterial activity demonstrated by marine algal extracts *in vitro* and in pilot food studies provides a basis for further research and product development in this area. The high efficiency of bacterial inhibition in comparison to industry standard preservatives discussed in this review makes algal extracts excellent candidates for incorporation into foods. Seaweeds are also non-toxic, inexpensive, and contain minerals, vitamins, and proteins.

## 7. Synergistic Effects

Synergism can occur between two or more compounds with pharmacological activity, such as orthodox drugs or natural products. This can produce a negative effect in the form of contraindication, where active substances exert a more potent effect when combined than they would if used individually. However, controlled synergistic interaction allows lower doses of the active substances to be used, which can result in fewer side effects [131]. Some marine algal derivatives have been reported to enhance the pharmacological activity of drugs used to combat human pathogenic bacteria. Lee *et al.* [132] examined the synergistic effect of combining the sulphated polysaccharide fucoidan from brown seaweeds with antibiotics to inhibit cariogenic and periodontopathogenic bacteria which cause dental infections. The fucoidan (analytical grade) was combined with either ampicillin or gentamicin, two common antibiotics. The minimum inhibitory and bactericidal concentrations of the fucoidan/antibiotic complexes were measured against *Streptococcus* *mutans*, *Streptococcus* *sanguinis*, *Streptococcus* *sobrinus*, *Streptococcus* *ratti*, *Streptococcus* *criceti*, *Streptococcus* *anginosus*, *Streptococcus* *gordonii*, *Actinobacillus actinomycetemcomitans*, *Fusobacterium nucleatum*, *Prevotella intermedia*, and *Porphylomonas gingivalis*. The results were highly significant. The MIC and minimum bactericidal concentration (MBC) values were reduced on average by a factor of four. Therefore, only a quarter of the ampicillin or gentamicin dose was required to kill all species of bacteria when combined with fucoidan. The study concluded that the inherent antibacterial properties of fucoidan, and its ability to affect bacterial cell wall synthesis, was responsible for the synergistic effect observed. The formation of bacterial biofilms in the mouth commonly leads to dental cavities, gingivitis, and periodontitis. If antibiotics must be prescribed for a patient, a significant reduction in the required dose such as observed in the above study would be beneficial for the individual’s gut health and immune system.

He *et al.* [133] also reported a synergistic effect using marine algal derivatives and antibiotics. In the study, the antibiotic azithromycin was combined with a marine alginate-derived oligosaccharide (ADO). MIC values required to inhibit wild-type resistant *Pseudomonas aeruginosa* were measured for azithromycin, and for the azithromycin/ADO complex. The MIC value was significantly reduced by a factor of 2.8 by the addition of the ADO. The MIC for azithromycin alone was 152.6 μg/mL. This was reduced to 54.2 μg/mL using the azithromycin/ADO complex. This synergistic effect was attributed to the ability of charged groups in ADO to interact with anionic/cationic groups on the bacterial cell surface, forming a layer that blocks the transport of essential materials into the cell. In addition, G subunits of ADO chelate cations such as Ca^2+^ thereby acting as an antagonist toward voltage-operated calcium channels. Quorum sensing-controlled virulence factors (elastase B and pyocyanin) and biofilm development was also suppressed by the addition of ADO, allowing the reduced level of azithromycin to act more effectively. Synergism such as this between marine algal derivatives and traditional antibiotics continues to be developed as an innovative approach to combat antibiotic-resistant bacteria.

## 8. Biotechnological Potential of Marine Algae

Algal biotechnology entails the use of micro or macroalgae to produce or transform useful products for specific functions [134]. This may involve manipulating the algal cells into overexpressing a secondary metabolite, or some compound of pharmacological or industrial interest by altering environmental parameters such as temperature, pH, or food availability. Using an organism as a cell factory in this manner has been successfully applied to bacteria, plants, and many other organisms for decades. The microalga, *Dunaliella* *salina*, for example, naturally produces high concentrations of β-carotene to survive its hypersaline environment. Controlling salinity in bioprocessors containing *Dunaliella* *salina* forces the alga to increase β-carotene synthesis which can then be harvested for human use [135]. Biotechnology may also involve transgenesis, which is the introduction of a foreign (*trans*) gene into a cell in order for the organism to produce a new, inheritable trait such as the synthesis of a new biomolecule or expression product. Genes can be introduced to algal cells to produce biomolecules with antibacterial activity such as phlorotannins or terpenes. A recent study by Guzmán-Zapata *et al.* [136] described the successful, inheritable genetic transformation of chloroplasts in the green microalga *Chlamydomonas reinhardtii* to produce useful recombinant proteins.

In addition, marine algal antibacterial compounds, such as polysaccharides, are currently being studied for their nanobiotechnological applications in drug delivery, wound dressing, cancer therapy, and tissue engineering. Their biocompatibility, biodegradability, and lack of toxicity makes them promising leads in this area [137].

### Omics Studies

More recently developed biotechnological tools such as transcriptomics, nutrigenomics, metabolomics, proteomics, and metagenomic profiling are now being applied to algae, and will aid in the understanding of their genome and their pharmacological interactions with bacteria [138,139,140]. For example, transcriptomic studies on marine algae provide useful genomic data that may be used to identify species with antibiotic potential. The transcriptome is the set of all messenger RNA molecules in one cell or population of cells. Sequencing the transcriptome allows for the comparison and identification of genes that are differentially expressed in discrete cell populations, or in response to different environmental factors [141,142]. Hovde *et al.* [143] sequenced the transcriptome of the microalga, *Chrysochromulina tobin* at seven time points over a 24h light/dark circadian cycle. Significant differences in gene expression were seen at different time points for processes such as fatty acid synthesis and modification. Amongst the genes identified, those involved in defence of the alga were found to encode potential antibiotics, antibiotic extrusion proteins, and novel antibacterial peptides. The transcriptomic sequencing also revealed the first algal polyketide synthase-non ribosomal peptide synthetase. The findings may provide potential routes for the synthesis of therapeutics and useful novel metabolites. Algal compounds with potential pharmacological activities were also reported by de Oliveira *et al.* [144] from the transcriptomic sequencing of the red seaweed, *Laurencia dendroidea*, and its microbiome. Six specimens of *Laurencia dendroidea* and their transcriptomes were sequenced from three Rio de Janeiro coastal locations. Genetic sequences related to terpene biosynthesis and the mevalonate-independent pathway were identified. Understanding the genomic mechanisms of these pharmacologically active secondary metabolites provides a potential for novel biotechnological applications.

## 9. Chemical Synthesis

A number of challenges exist for the development of marine algal antibacterial products, such as supply, sourcing, and ecological issues. This is particularly applicable to compounds with pharmacological activity that are present in miniscule quantities within a microalga or seaweed. Mariculture of selected species is an option, however it is labour intensive and would still necessitate the production of large volumes of biomass. The marine environment in which an alga of interest grows can be very inaccessible, and incur huge harvesting risks and costs [145]. It would also be ecologically damaging to remove substantial quantities of any species from their natural marine, or other, environment. Total chemical synthesis of pharmacologically activity natural compounds offers a solution. Chemical synthesis of rare natural drug compounds has been used successfully for many terrestrial plants. An example is the synthetic anticancer drug paclitaxel, which was developed from taxol, a naturally occurring extract of Pacific yew (*Taxus brevifolia*) bark. This rare tree would have become extinct if harvested continuously. However, its chemical structure was elucidated, with the synthetic form exerting an identical mechanism of action on cancer cells. Several natural marine products have been successfully synthesised, such as the analgesic ziconotide (Prialt). This is the synthetic form of ω-conotoxin peptide, originally derived from a defence toxin produced by the marine Cone Snail. Urosa *et al.* [146] synthesised Luffarin I, a sesterterpenolide, originally found in the sea sponge, *Luffariella geometrica*, using commercially available sclareol. The chemical synthesis of marine algal antibacterial compounds is now a growing area of research. The genetic mechanism of terpene biosynthesis in the red seaweed, *Laurencia dendroidea*, was recently elucidated by de Oliveira *et al.* [147]. This genus of seaweed produces halogenated terpenoids and acetogenins which have important biotechnological applications, however, little was known about the genes that code for these compounds. The study identified forty one genes involved in the biosynthesis of terpenoid precursors and terpene synthases. The findings pave the way for the chemical synthesis of terpenes with antibacterial and other applications.

Total chemical synthesis of bioactive compounds such as algal antibacterials has many advantages aside from increased supply. It protects the marine environment from over-harvesting and allows for research into chemical structure-activity relationships and drug lead optimisation of the antibacterial. Modifications of the structure, such as the addition of polar or non-polar functional groups, can lead to increased efficacy at the target site, reduced side effects, and improved physiochemical and metabolic properties [148,149].

## 10. Conclusions

Drug-resistant bacteria pose an increasing challenge to global health. The European Centre for Disease Prevention and Control estimates that antimicrobial resistance results in over 25,000 deaths across Europe each year [150]. Antibacterial agents with pharmacological mechanisms of action that differ to those of traditional antibiotics must be developed to stem the rise in global bacterial resistance. The *in vitro* studies discussed in this review show promise for similar *in vivo* efficacy. Some hurdles exist in marine product development, such as supply issues, determination of the efficacy target, and full chemical synthesis. As with all areas of drug discovery, extensive clinical trials will be required to determine the *in vivo* fate of marine antibacterial extracts on mammalian cells in terms of first pass metabolism and possible toxicity. The marine environment is home to an immense taxonomic diversity that has remained relatively unexplored in drug discovery by terrestrial standards. In order to overcome the challenges to marine natural product development a multi-disciplinary strategy can be adapted which utilises nascent technologies and tools for developing novel antimicrobial agents.
